# Videoendoscopy of Colonic Early Cancer

**DOI:** 10.1155/DTE.1.125

**Published:** 1995

**Authors:** Yoshihiro Sakai

**Affiliations:** Division of Digestive Endoscopy Toho University Ohashi Hospital 2-17-6 Ohashi Meguro-ku Tokyo 153 Japan

## Abstract

Since January 1982, 275 early colonic carcinomas undergoing total endoscopic resection were studied. Of this series, 234 lesions showed some adenoma components, whereas the remaining 41 lesions lacked adenoma components. Minute carcinomas measuring ≤ 5 mm (21 lesions) were most commonly the hemispheric protruding type lesions (IIs, 9 lesions), followed by superficial protruding type lesions with a height of ≤ 3 mm (IIa, 6 lesions). There also were 2 superficial depressed-type lesions that were slightly concave. Eight of these minute carcinomas may have developed by de novo carcinogenesis, and 2 had already invaded the submucosa. Therefore every effort should be made not to overlook lesions measuring ≤ 5 mm in diameter. That none of these lesions were located in the rectum indicates the acute necessity for improved examination procedures. With regard to IIa lesions measuring > 5 mm, 92.3% were discovered by videoendoscopy. This high detection rate was attributed to the growing use of videoendoscope systems and reflects heightened interest on the part of endoscopists in superficial type lesions.


*Diagnostic and Therapeutic Endoscopy*, 1995, Vol. 1, pp. 125–130
Reprints available directly from the publisher
Photocopying permitted by license only

© 1995 Harwood Academic Publishers GmbH
Printed in Malaysia

### Videoendoscopy of Colonic Early Cancer

YOSHIHIRO SAKAI

*Division of Digestive Endoscopy, Toho University Ohashi Hospital, 2-17-6 Ohashi, Meguro-ku, Tokyo 153, Japan*

*(Received November 1, 1993; in final form, January 22, 1994)*

Since January 1982, 275 early colonic carcinomas undergoing total endoscopic resection were studied. Of this series, 234 lesions showed some adenoma components, whereas the remaining 41 lesions lacked adenoma components. Minute carcinomas measuring ≤ 5 mm (21 lesions) were most commonly the hemispheric protruding type lesions (IIs, 9 lesions), followed by superficial protruding type lesions with a height of ≤ 3 mm (IIa, 6 lesions). There also were 2 superficial depressed-type lesions that were slightly concave. Eight of these minute carcinomas may have developed by de novo carcinogenesis, and 2 had already invaded the submucosa. Therefore every effort should be made not to overlook lesions measuring ≤ 5 mm in diameter. That none of these lesions were located in the rectum indicates the acute necessity for improved examination procedures. With regard to IIa lesions measuring > 5 mm, 92.3% were discovered by videoendoscopy. This high detection rate was attributed to the growing use of videoendoscope systems and reflects heightened interest on the part of endoscopists in superficial type lesions.

**KEY WORDS:** colonic early cancer, minute carcinoma

### INTRODUCTION

The approximately 10-year period since videoendoscopes have become commercially available has witnessed significant improvements in endoscopic instrumentation, resulting in enhanced diagnostic capability, maneuverability, and image recording quality. Despite product enhancement, videoendoscopes still do not possess clear-cut advantages over fiberscopes, because the functions of fiberscopes have been transferred to videoendoscopes and, conversely, technologic improvements obtained during the development of videoendoscopes have been incorporated into fiberscopes. It must be conceded that videoendoscopes relieve the endoscopist of having to having to stare at bright images with a single eye and do not require continual adjustment of the endoscopist's visual acuity. Videoendoscopes also do not require the user to maintain a bent-over posture and are lighter than fiberscopes, because they do not have an eye lens, thereby increasing maneuverability and reducing operator fatigue (1). By connecting the system to a TV monitor, multiple images can be displayed simultaneously to ensure accurate diagnosis and reduce the risk of overlooking clinically significant lesions.

Although comparable capabilities can be obtained by connecting a fiberscope to an endoscopic TV System, performance is compromised. Videoendoscopes excel in image-filing capability and reproducibility of VTR recordings. They also offer excellent image processing by permitting outline enhancement and hue adjustment. By overcoming the critical flaw of conventional endoscopes with poor outline enhancement capability (i.e., image flattening caused by the close proximity of the illumination lens and the objective lens), videoendoscopes provide sharp image definition and hue contrast in cases with slight depressions or elevations, thereby facilitating the detection and differential diagnosis of abnormalities.

### CLASSIFICATION OF EARLY CANCER

Early cancer of the colon and cofined to mucosa and is not associated with any lymph node metastases confined to the mucosa. The General Rules for Clinical and

Address for correspondence: Yoshihiro Sakai, M.D., Division of Digestive Endoscopy, Toho University Ohashi Hospital, 2-17-6 Ohashi, Meguro-ku Tokyo 153.

Pathologic Studies on Carcinoma of the Colon (2) therefore classify lesions with invasion confined to the submucosa and without as early colonic cancer. Based on these criteria, the author studied 275 lesions that were totally resected endoscopically and histologically diagnosed to be colonic cancer during the period from January 1982 to August 1993. All of these carcinomas were confined to the mucosa or to the submucosa (Table 1).

In this series, 234 lesions showed some adenoma tous components, whereas the remaining 41 lesions consisted of only cancer tissue. The longest dimension of the lesion was ≤ 5 mm, > 5 to ≤ 10 mm, and > 10 mm in 21 lesions, 78 lesions, and 176 lesions, respectively. Although the majority of lesions (64%) were > 10 mm, the remaining 36% measured ≤ 10 mm, and 7.6% of the total were ≤ 5 mm. Histologically, although the possibility cannot be ruled out that cancer cells may have replaced adenoma cells during the development of the 33 lesions without adenomatous components that measured > 5 mm, the remaining 8 lesions without adenomatous components that were ≤ 5 mm in diameter most likely resulted from *de novo* carcinogenesis. It should also be noted that these 8 lesions accounted for 19.5% of the 41 lesions without adenomatous components. This figure is far higher than the 5.5% (13/234) of the lesions measuring ≤ 5 mm with adenomatous components. Detection of carcinomas without adenoma components that measure < 5 mm is therefore of the utmost importance clinically.

### DIAGNOSIS OF MINUTE CARCINOMAS

The 21 lesions measuring ≤ 5 mm, defined as minute carcinomas (3,4) were classified by location and macroscopic type to delineate their characteristics (Table 2). Location was expressed according to the anatomic divisions of the colon. Lesions situated in the sigmoid colon (S) and descending colon (D) were classified together, as were those located in the ascending colon (A) and cecum (C). None of the lesions were found in the rectum (R). Although 11 lesions were situated in the sigmoid or descending colon, 8 were located in the transverse colon (T), making this the most common single segment for the development of minute carcinomas.

**Table 1** Size and histologic characteristics of endoscopically resected early carcinomas

| | *≦ 5* | *5 < ≦ 10* | *10 mm<* | *Total* |
|---|---|---|---|---|
| Ca $\bar{c}$ ad | 13 | 69 | 152 | 234 |
| Ca $\bar{c}$ ad | 8 | 9 | 24 | 41 |
| | 21 | 78 | 176 | 275 |

Note.—Ca $\bar{c}$ ad, indicates cancer with adenoma;

Ca $\bar{c}$ ad, cancer without adenoma.

**Table 2** Location and macroscopic type of microcarcinomas ≤ 5 mm in diameter

| | *Ip, Isp* | *Is* | *II a (+ II c)* | *II c* | *Total* |
|---|---|---|---|---|---|
| R | | | | | |
| S, D | 3 (2) | 4 (2) | 3 | 1 (1) | 11 (5) |
| T | 1 | 3 | 3 (1) | 1 (1) | 8 (2) |
| A, C | | 2 (1) | | | 2 (1) |
| | 4 (2) | 9 (3) | 6 (1) | 2 (2) | 21 (8) |

Note.—( ) indicates Ca $\bar{s}$ ad.

The macroscopic type was classified, according to the General Rules for Clinical and Pathologic Studies on Carcinoma of the Colon, into the protruding type (I) or superficial type (II) (Fig. 1). Protruding type lesions were further subdivided into pendunculated lesions (Ip) and nonpendunculated (sessile) lesions (Is). Semipendunculated lesions (Isp) were included in Ip. Superficial type lesions were divided into superficial elevated type ≤ 3 mm in height (IIa) and superficial depressed type (IIc). IIa lesions with a depressed center (IIa + IIc) were included in IIa. No superficial flat type lesions (IIb) were present in this series. Is lesions were most commonly encountered (9 lesions, 42.9%), followed by IIa (+IIc) and Ip/Isp. IIc lesions were the most rare.

No particular correlation was noted between location and macroscopic type.

The histologic features of these microcarcinomas also were studied by macroscopic type. Only 1 lesion, found in the sigmoid colon, of the 4 Ip/Isp lesions was distinctly pendunculated, whereas the other 3 lesions, presenting with narrowing at their bases, were classified as Isp. Two of these latter 3 lesions did not show any adenomatous components, and 1 had already invaded into the submucosa, although the degree of invasion was slight. Three of the 9 Is lesions lacked adenomatous components. One of the 6 IIa lesions and both of the IIc lesions similarly lacked adenoma components; 1 of these latter lesions showed slight invasion of the submucosa.

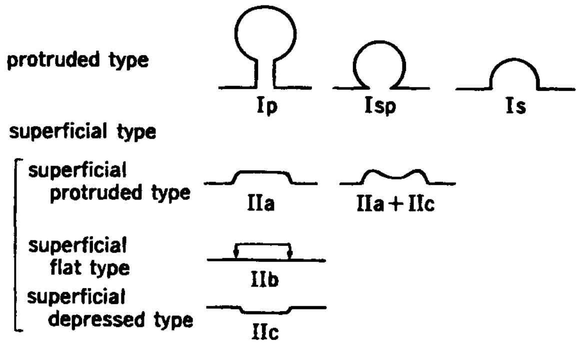

**Figure 1** Schema of macroscopic types of early colonic cancer.

Because routine examinations do not permit lesions with adenomatous components to be distinguished from those without such components in cases of minute carcinomas measuring ≤5 mm, a magnifying endoscope should be employed to determine the presence or absence of marginal adenoma tissue.

### DIAGNOSIS OF IIa LESIONS OF > 5 MM

No or minimal differences between the hue of low lesions and that of surrounding normal mucosa make endoscopic detection a challenging task. By defining superficial elevated lesions (IIa) as carcinomas measuring ≤3 mm in height with a border that is distinctly elevated from the surrounding mucosa on dye examination, 26 such lesions with maximum diameters of >5 mm were included in this series (Table 3). These lesions most commonly were located in the sigmoid colon and descending colon (8 lesions), followed by the ascending colon and cecum (7 lesions). The transverse colon was the most common single portion of the colon for lesions of this type. However, 5 of these lesions were found in the rectum.

Lesion dimension was categorized in 5-mm intervals. Lesions measuring > 5 to ≤ 10 mm were most frequently encountered (11 lesions). There were 5, 4 and 6 lesion with diameters of > 10 to ≤ 15 mm, > 15 to ≤ 20 and > 20 mm, respectively. There was no correlation between lesion location and size.

Only 2 of these 26 lesions were discovered by fiberscopes, and all other 24 remaining lesions were found by videoendoscopes.

### DISCUSSION

Since the introduction of a videoendoscope system in October 1986, about 8,000 endoscopic examinations have been performed as of August 1993. Videoendoscope systems have been used in approximately half of these examinations. Recent trends indicate that videoscopes currently are used in about 80% of patients who undergo endoscopy. Initially, the videoendoscope was restricted by the limited availability of examination rooms able to accommodate the bulky ancillary equipment. More recently, videoendoscope use has been steadily increasing owing to the more widespread availability of videoscope systems and because of its popularity as a result of the reduced fatigue for the endoscopist.

**Table 3** Location and size of IIa lesions >5 mm in diameter

| | 5< ≦10 | 10< ≦15 | 15 mm< | *Total* |
|---|---|---|---|---|
| R | 3 | 1 | 1 | 5 |
| S, D | 4 | 1 | 3 | 8 |
| T | 2 | 1 (1) | 3 | 6 (1) |
| A, C | 2 (1) | 2 | 3 | 7 (1) |
| | 11 (1) | 5 (1) | 10 | 26 (2) |

Note.—( ) indicates found by fiberscope.

Increased use of videoendoscopes also has been promoted by the belief that the "modified two-man technique" (5) using a videoendoscope is an effective training exercise for introductory programs in colonoscopy. In the modified two-man technique, colonoscopy is performed by two physicians: a highly experienced endoscopist and an endoscopist in training. The less experienced endoscopist usually serves as assistant in the initial phase of training. The roles are then reversed, and the experienced endoscopist acts as assistant and instructs the younger endoscopist in the direction of tip flexion and teaches the effects of insufflation and aspiration. The senior endoscopist is responsible for rotating the scope, guiding it to the cecum, and removing it, while ensuring minimal discomfort to the patient. To accomplish this procedure successfully it is far more advantageous to use a large TV monitor than a fiberscope combined with a lecturescope or teaching instrument. Moreover, after the operator becomes accustomed to the equipment, tip angulation can be controlled with the left hand alone, leaving the right hand unencumbered to carry out the tasks of the assistant. The entire procedure therefore can be performed by a single endoscopist. This is referred to as the "one-man technique." The modified two-man technique allows an endoscopist to develop and make a smooth transition to the one-man technique.

Another reason that videoendoscope systems are preferred is the reduced time required for endoscopic therapeutic procedures that results when an assistant is able to manipulate forceps or snares while viewing the same image as the operator. Videoendoscopes are also equipped with a freeze function that permits target images to be selected as often as desired. This function eliminates image distortion caused by bubbles on the lens or rapid movement of moving images. Younger endoscopists therefore tend to use videoendoscope systems because of the low risk of failure in image viewing and recording. Consequently, fiberscopes increasingly tend to be used only for screening or as a last resort if a videoendoscope is not available.

Massive lesions protruding into the lumen of the colon can be diagnosed easily with either a fiberscope or a videoendoscope. This also applies to small lesions that protrude into the lumen or lesions with a color distinct from the surrounding mucosa. Even low lesions that are large or are different in color from the mucosa most likely will be detected by either type of scope. However, there is a high risk of overlooking small, low lesions that are the same color as surrounding mucosa (6). Likewise, lesions that are situated on the oral side of taut mucosal folds or lesions that can be viewed directly but have been overdistended by excessive insufflation may be overlooked. This also applies in the event of inadequate pretreatment.

It is impractical to analyze factors leading to the discovery of lesions. Although the percentage of IIa lesions discovered with videoendoscopes is high, the aforementioned continual evolution of scopes and techniques for their use precludes unconditional recommendations for one type of endoscope or the other. In cases where endoscopy is used to evaluate lesions already detected by barium enema, a videoendoscope generally is selected because it facilitates the execution of therapeutic procedures. However, that only 2 of the 26 IIa lesions >5 mm were detected by fiberscopy, whereas the other 24 lesions where discovered by videoendoscopy, indicates that lesions of this type are easier to detect with a videoendoscope.

Apart from protruding type lesions (I), superficial type lesions (II) were characterized by minimal differences in color tone from the surrounding mucosa (Fig. 1A), slight semilunar fold deformity, or, in rare instances, an abnormal pattern of illuminated reflection points on the mucosa. Other abnormalities include interrupted course of blood vessels or an unnatural luster of the mucosal surface (Fig. 2A). These changes can be overlooked easily but on close inspection, they can be identified as signs of abnormalities (Fig. 1B). Definition of the extent of some lesions may require dye spray (Fig. 2B). Spraying 10 to 20 ml of 0.05% methylene blue solution through the biopsy channel can help demarcate the borders of lesions.

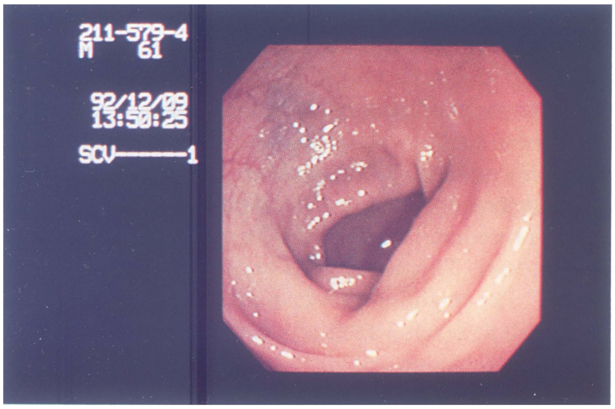

**Figure 1A** A microcarcinoma appears as a reddish spot.

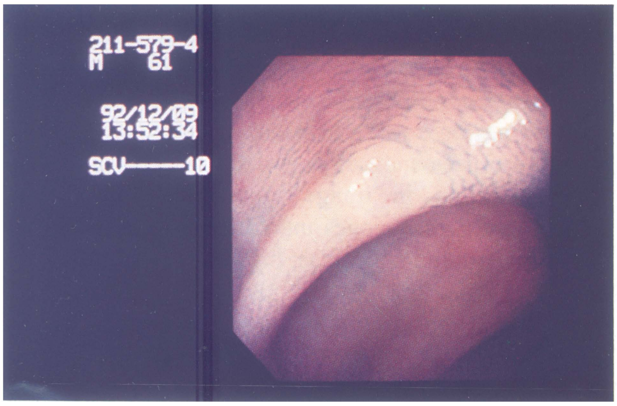

**Figure 1B** Close-up view of the same lesion after dye injection. The dye was retained in the center of the lesion, demonstrating the reddish region to be depressed. Cancer was present in only the depressed region (2mm).

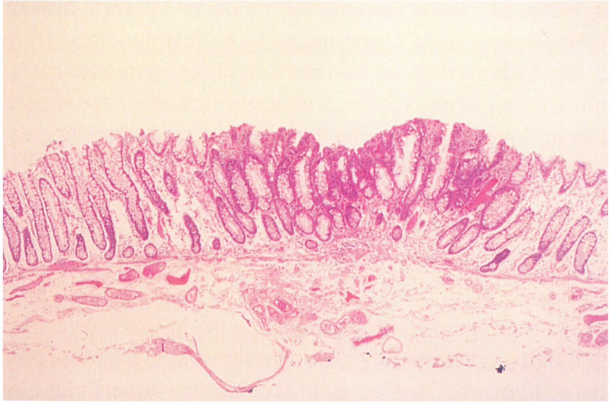

**Figure 1C** Microscopic findings of the endoscopically resected specimen.

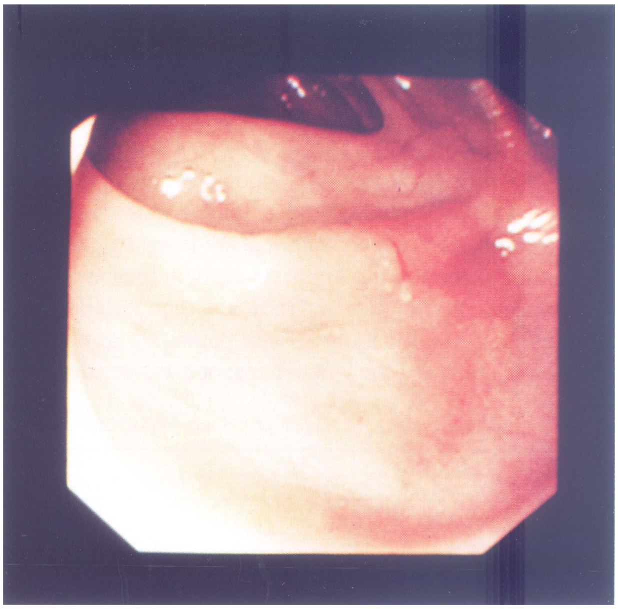

**Figure 2A** A superficial elevated type carcinoma, appearing as indistinctly pigmented patches by conventional observation.

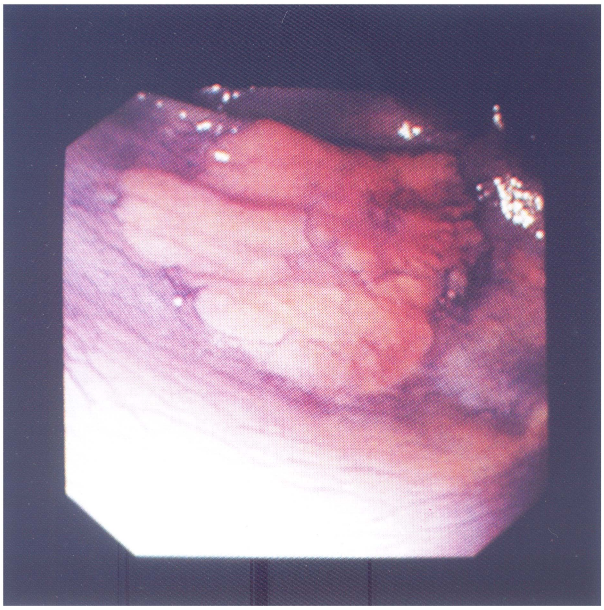

**Figure 2B** The same lesion directly after dyeing. The outline of the lesion became distinct.

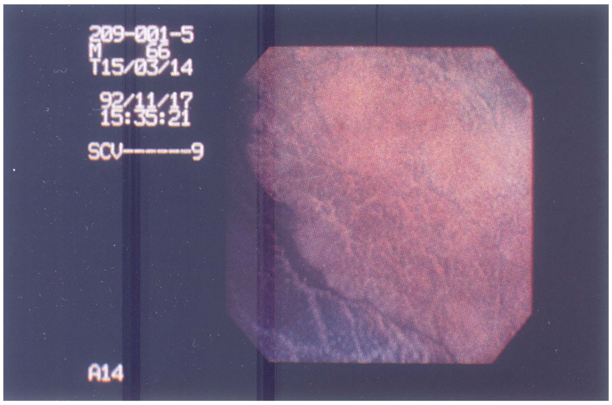

**Figure 2C** A portion of the same lesion magnified 30x.

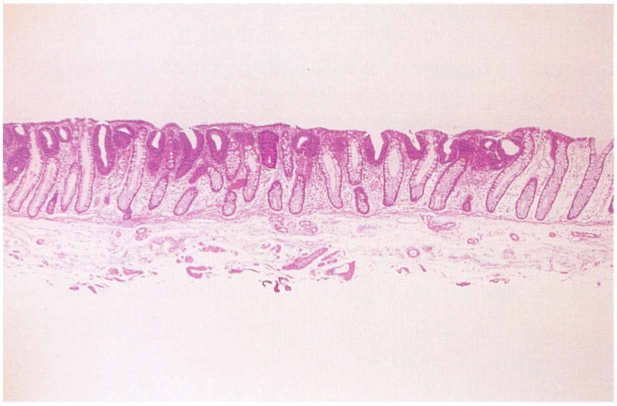

**Figure 2C**

By aspirating off the excess dye and washing the mucosa, the outline of the lesion often can be examined in detail. Scopes with zoom-type magnification capability can be used to observe the shape and arrangement of small ducts (Figure 2C).

Despite the fact that no microcarcinomas were detected in the rectum, they were frequently noted in the transverse colon. This significant difference in the distribution of lesions from that generally found for carcinomas treated surgically probably indicates a problem in examination methods. Because it is necessary to prevent excess air insufflation when inserting the scope, observation of the proximal colon was not adequate. Because examination of the colon generally is initiated after arriving at the rectum and observing the terminal ileum, the surface of the mucosa is examined by inflating and deflating as the colon scope is being withdrawn. The rectum is therefore examined last when it is in the most distended state and thus potentially increases the risk of overlooking minute carcinomas in the colon. The relatively high number of minute carcinomas discovered in the transverse colon is apparently caused by this being a region that can be examined easily while either inserting or withdrawing the scope. For regions such as the rectum or sigmoid colon, which are associated with a high risk of cancer, dye spray should be used, even in the apparent absence of anomalies, to increase the possibility of detecting minute lesions. Because lesions > 6 mm in diameter, including difficult-to-detect lesions such as IIa carcinomas, can be diagnosed in these regions as in other segments of the colon, particular caution must be exercised with regard to minute carcinomas.

### ACKNOWLEDGMENTS

Part of this paper was presented at the International Symposium, "The Latest Frontiers of Endoscopy," on March 31, 1993 at the 79th General Meeting of The Japan Society of Gastroenterology. The authors are grateful to Professor J. Patrick Barron of the International Medical Communications Center of Tokyo Medical College for his review of the manuscript.

### REFERENCES

1. Sakai Y, Fujinuma, S, & Ibe A. Electronic colonoscopy; Past and present, Endoscopia Digestiva 1989;1:469–476. (Japanese)
2. Japan Research Society for Cancer of Colon and Rectum. General rules for clinical and pathological studies on cancer of colon, rectum and anus, 4th ed. Kanahara-Shuppan, Tokyo, 1985. (Japanese)
3. Kudo S, Miura K, Takano Y, et al. Detection of colorectal minute cancer, Stomach and Intestine 1990;25:801–812. (Japanese)

4. Okamoto H, & Sasaki T. Detection and importance of colonic diminutive lesions (5mm or less in diameter), Stomach and Intestine 1990;25:813–818. (Japanese)
5. Vatanabe S, Ohashi S, Akiya M, et al. the educational utility of the modified two men method, Therapeutic Research 8 (suppl 1) 1988;239–242. (Japanese)
6. Katakura S, Satake Y, Aksoz K, et al. Endoscopic and histopathological study about 25 cases of minute superficial depressed neoplastic lesions in the large intestine, Digestive Endoscopy 1993;5:3–12

